# Supplementary material for: Miniaturised interaction proteomics on a microfluidic platform with ultra-low input requirements
Source: Nat Commun. 2019 Apr 4;10:1525. doi: 10.1038/s41467-019-09533-y (PMC6449397; doi:10.1038/s41467-019-09533-y)
Supplement: Supplementary file 1 — Supplementary Information [file 41467_2019_9533_MOESM1_ESM.pdf]

Supplementary Information

Miniaturised interaction proteomics on a microfluidics platform with ultra-low input requirements.

Furlan et al.

Supplementary Figure 1

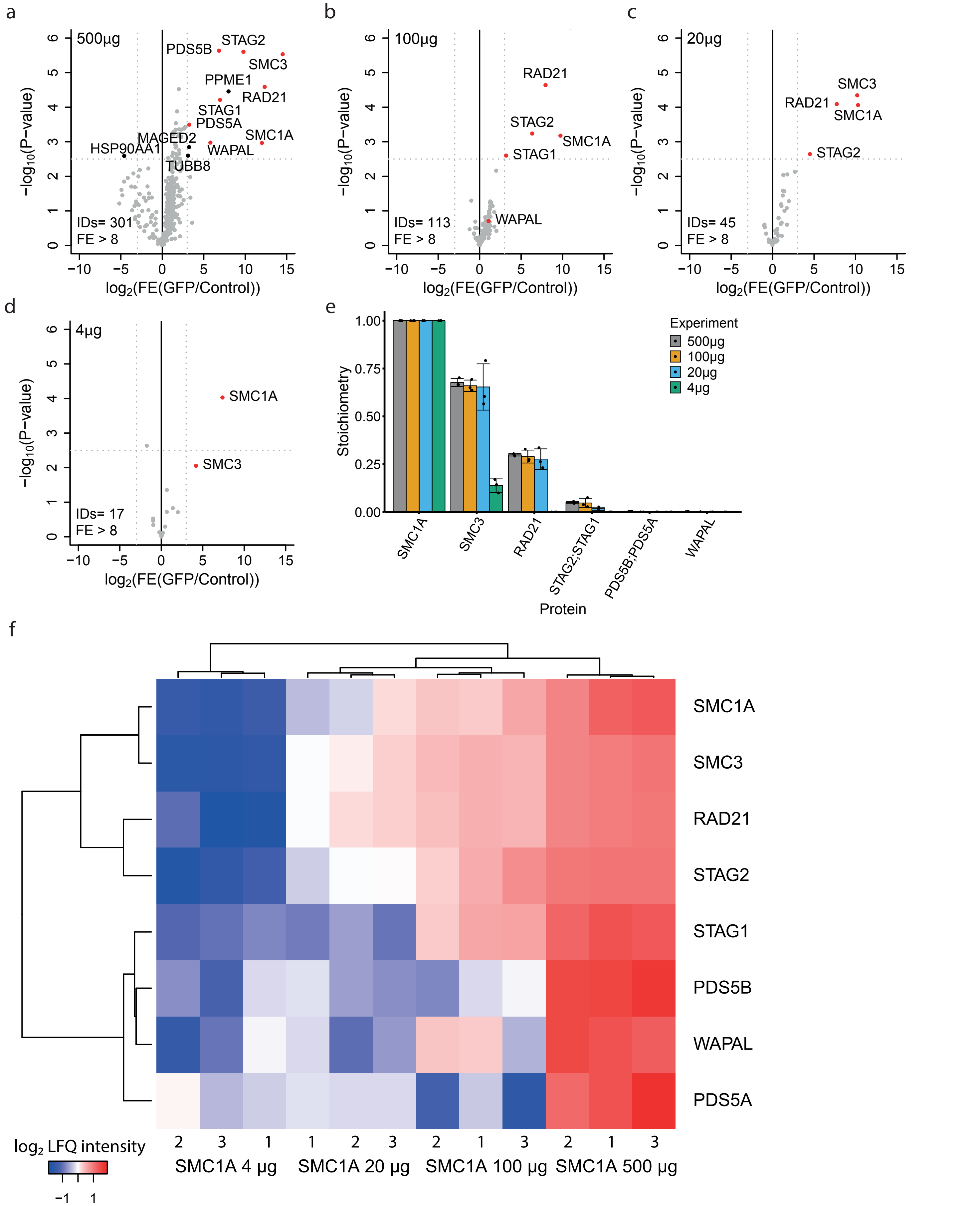

Supplementary figure 1 Sensitivity loss in the detection of interactions when using decreasing amounts of input material over same reaction volume in regular AP-MS. a–d Volcano plot of regular pull-down with 500 µg (a), 100 µg (b), 20 µg (c) and 4 µg (d) of total extract, respectively for SMC1A-GFP bait in 400 µL reaction volume. Dotted grey lines represent statistical cut-offs. Black dots identify significant proteins; red dots are used for known interactors of the bait. TUBB8 is a known contaminant of pull-downs[1]. Each volcano plot presents n=1 biologically independent experiment, each comprised of n=3 technical replicates for PI-GFP and control extract.
e Stoichiometry of cohesin complex relative to the SMC1A bait as for experiments a, b, c and d. iBAQ values for each of the interaction partners of Cohesin complex were divided by the iBAQ values of the bait (SMC1A, set to 1). Data display mean values ± s.d. Grey filled bars are used for Cohesin complex stoichiometry as calculated in the microcentrifuge tube-based pull-down using 500 µg input, yellow bars for 100 µg input, light blue bars for 20 µg input and green bars for 4 µg input. Black dots represent the individual data points. Source data are provided as a Source Data file. f Heatmap of log2 LFQ intensity for cohesin subunits. Hierarchical clustering performed using Manhattan distances metric and linkage complete criterion.

Supplementary Figure 2

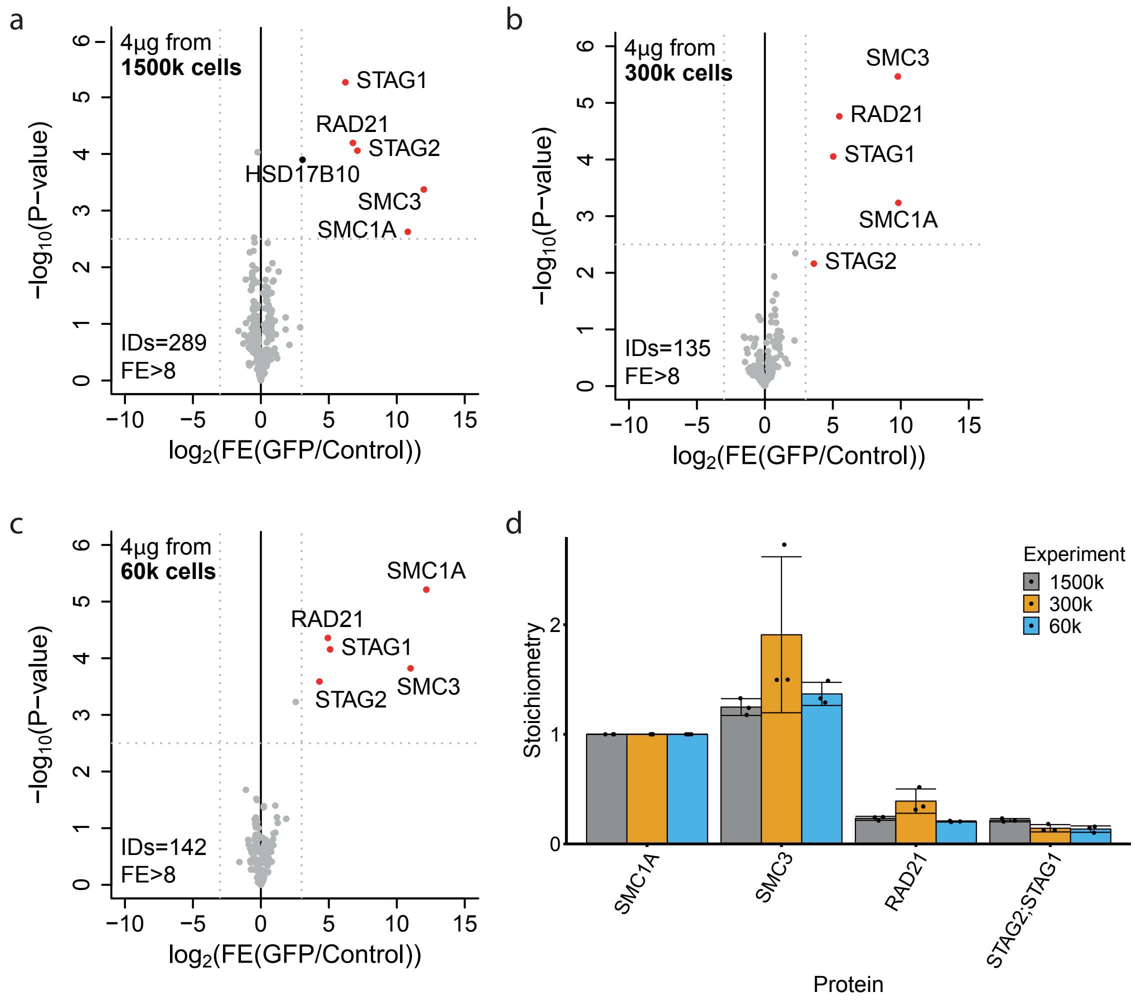

Supplementary figure 2 Initial material quantity for extract preparation does not affect detection of interaction partners of SMC1A. a—c Volcano plot from SMC1A on-chip AP-MS of 4 µg of extract prepared from 1.5 million cells (a), 300,000 cells (b) or 60,000 (c) cells respectively. Each volcano plot presents n=1 biologically independent experiment, each comprised of n=3 technical replicates for PI-GFP and control extract. d Stoichiometry of Cohesin complex components of the extracts presented in panels a—c as compared to the bait. iBAQ values for each of the interaction partners of Cohesin complex were divided by the iBAQ values of the bait (SMC1A, set to 1). Data display mean values ± s.d. Grey filled bars are used for Cohesin complex stoichiometry as derived by 1.5 million cells experiment, yellow bars for 300k cells experiment and light blue ones for 60k cells experiment. Black dots represent the individual data points. Source data are provided as a Source Data file.Source data are provided as a Source Data file.

Supplementary Figure 3

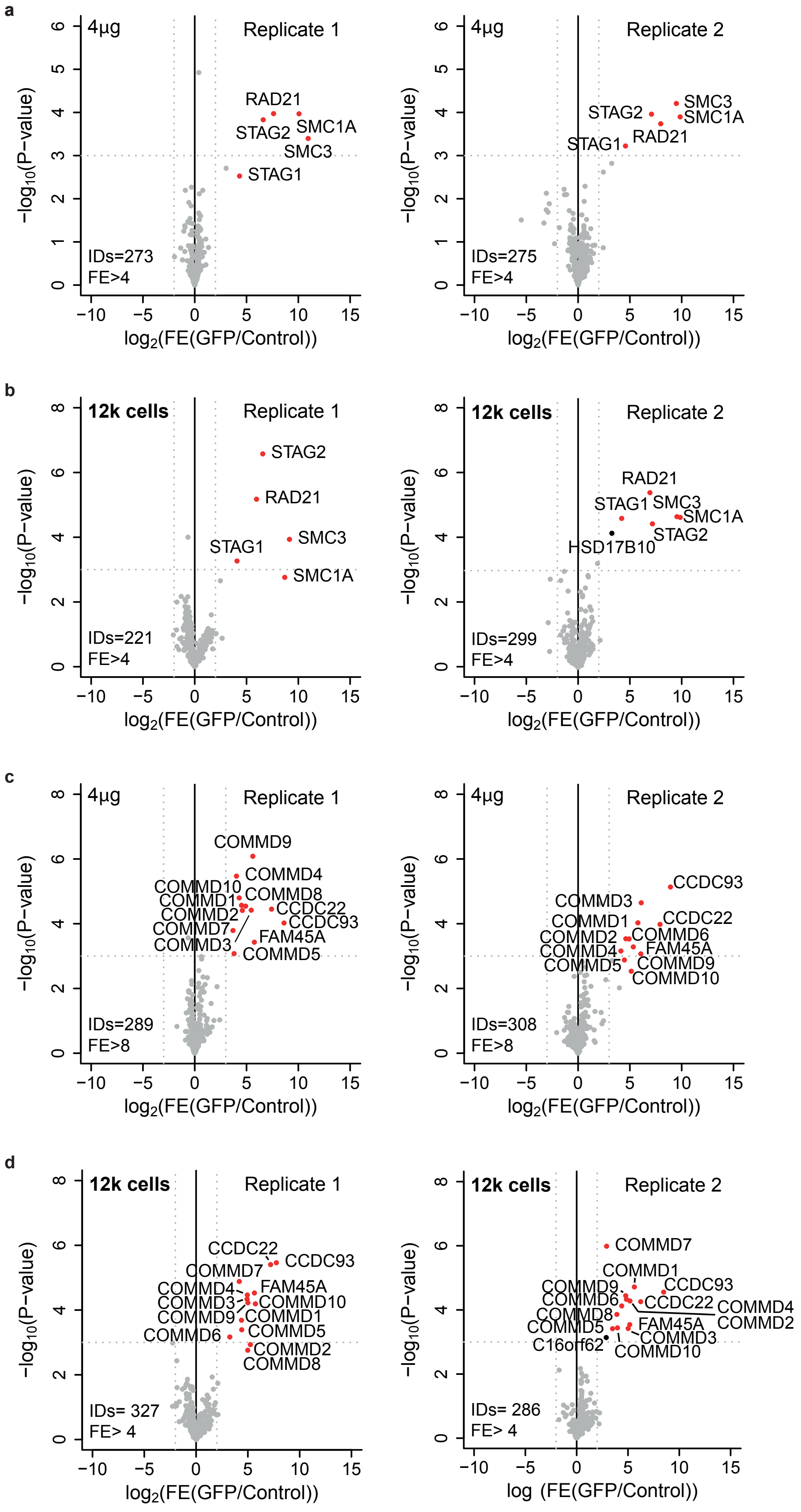

Supplementary figure 3 Biological and technical replicates for on-chip AP-MS.
a Volcano plots from individual experiments for on-chip AP-MS performed using 4 µg of total bulk protein extract from SMC1A-GFP and HeLa Kyoto control cells. b Independent experiments performed on extract prepared from single aliquots of 12,000 cells of SMC1A-GFP and HeLa Kyoto control cells. c, d as in panel a, b but utilizing CCDC93-GFP and HeLa Kyoto control cells.
Each volcano plot represents n=3 technical replicates. Red dots show proteins belonging to the specific complex.
Dotted grey lines represent statistical cut-offs. Black dots identify significant proteins; red dots are used for known interactors of the bait.
ID refers to the total number of proteins reported and FE stands for fold enrichment.

Supplementary fig. 4

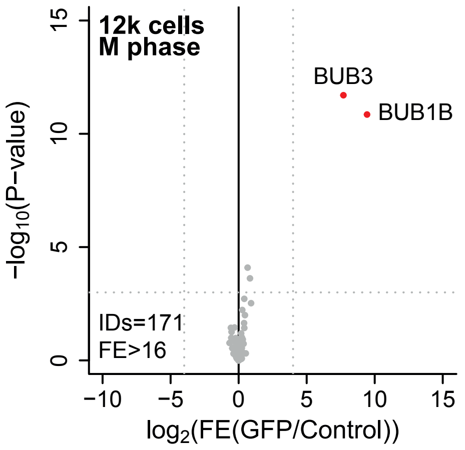

Supplementary figure 4 Purification of mitotic BUBR1 complex.
Volcano plot of BUBR1 (BUB1B)-GFP pull-down performed on 12,000 mitotic cells obtained by cell sorting based on DNA content. Dotted grey lines represent statistical cut-offs. Black dots identify significant proteins; red dots are used for known interactors of the bait. The volcano plot represents n=2 biologically independent experiments using n=3 technical replicates for BUBR1-GFP (induced cells) and control extract (not induced cells). ID refers to the total number of proteins reported and FE stands for fold enrichment.

Supplementary Figure 5

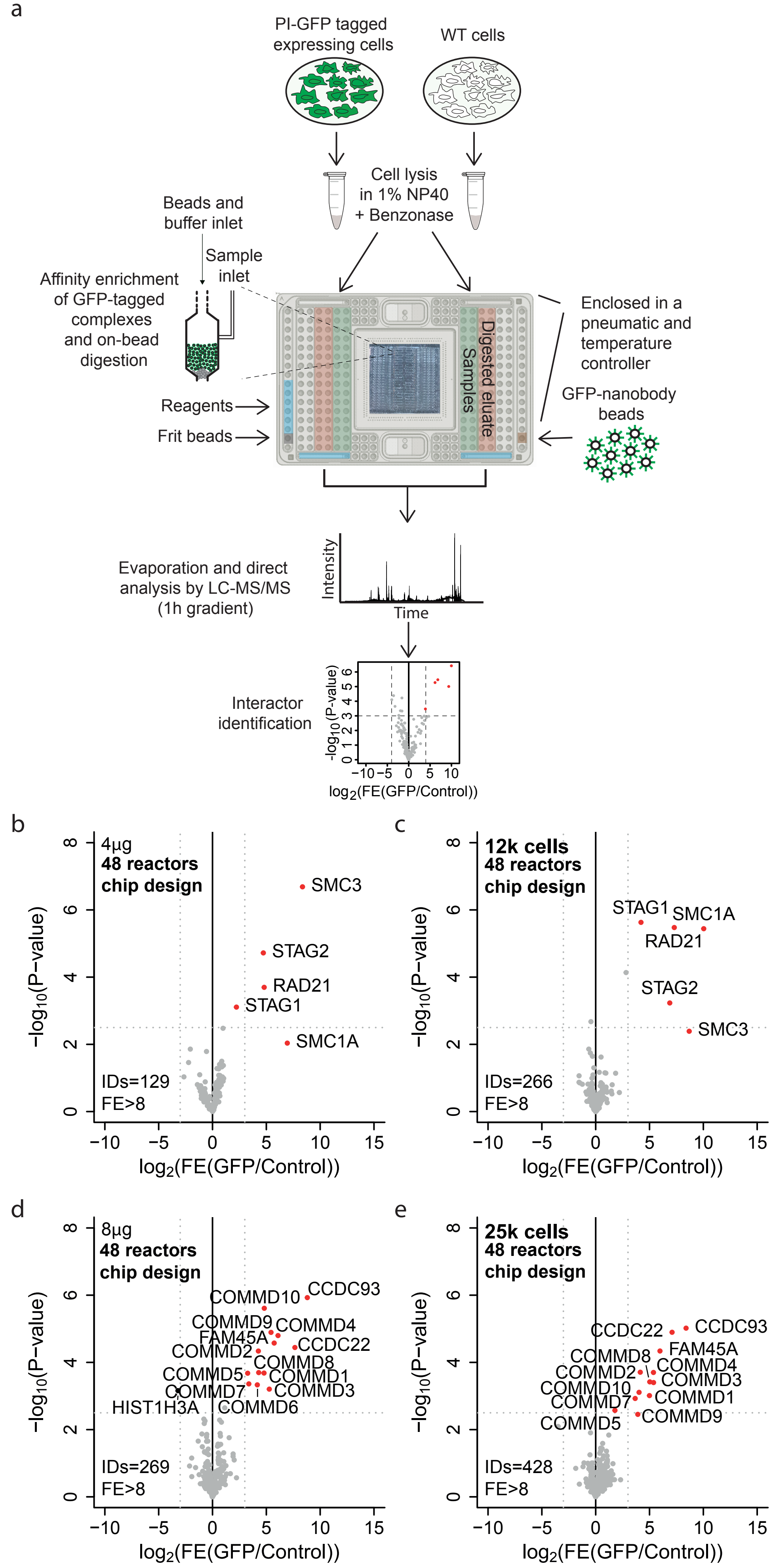

Supplementary figure 5 AP-MS on the 48 reaction chip.
a Workflow of on-chip AP-MS on a 48 reactors optimized microfluidics chip. b, c Volcano plots from SMC1A on-chip AP-MS of 4 µg of extract (b) or 12,000 cells (c). d, e CCDC93 on-chip AP-MS using 8 µg of lysate (d) or protein extract from 25,000cells (e).
Dotted grey lines represent statistical cut-offs. Black dots identify significant proteins; red dots are used for known interactors of the bait. Each volcano plot represents n=3 technical replicates. ID refers to the total number of proteins reported and FE stands for fold enrichment.

Supplementary References

1. Mellacheruvu, D. et al. The CRAPome: A contaminant repository for affinity purification-mass spectrometry data. Nat. Methods 10, 730–736 (2013).
