# Supplementary material for: Miniaturised interaction proteomics on a microfluidic platform with ultra-low input requirements
Source: Nat Commun. 2019 Apr 4;10:1525. doi: 10.1038/s41467-019-09533-y (PMC6449397; doi:10.1038/s41467-019-09533-y)
Supplement: Supplementary file 3 — Description of Additional Supplementary Files [file 41467_2019_9533_MOESM3_ESM.docx]

**Description of Additional Supplementary Files**

**File Name: Supplementary Data 1**

**Description:** Protein groups file, filtered data (processed in R and Perseus), iBAQ values used for stoichiometry estimation and stoichiometry calculations for 500 µg, 100 µg, 20 µg and 4 µg input extract microcentrifuge tubes-base SMC1A GFP pull-down.

**File Name: Supplementary Data 2**

**Description:** Protein groups file, filtered data (processed in R and Perseus), iBAQ values used for stoichiometry estimation and stoichiometry calculations for 4 µg input extract on-chip SMC1A GFP pull-down.

**File Name: Supplementary Data 3**

**Description:** Protein groups file, filtered data (processed in R and Perseus), iBAQ values used for stoichiometry estimation and stoichiometry calculations for 4 µg input extract derived from 1.5 million, 300 thousands and 60 thousands cells on-chip SMC1A GFP pull-down.

**File Name: Supplementary Data 4**

**Description:** Protein groups file, filtered data (processed in R and Perseus), iBAQ values used for stoichiometry estimation and stoichiometry calculations for 12 thousands cells input extract on-chip SMC1A GFP pull-down.

**File Name: Supplementary Data 5**

**Description:** Protein groups file, filtered data (processed in R and Perseus), iBAQ values used for stoichiometry estimation and stoichiometry calculations for 4 µg input extract on-chip CCDC93 GFP pull-down.

**File Name: Supplementary Data 6**

**Description:** Protein groups file, filtered data (processed in R and Perseus), iBAQ values used for stoichiometry estimation and stoichiometry calculations for 12 thousands cells input extract on-chip CCDC93 GFP pull-down.

**File Name: Supplementary Data 7**

**Description:** Protein groups file, filtered data (processed in R and Perseus), iBAQ values used for stoichiometry estimation and stoichiometry calculations for 25 thousands cells input extract on-chip CDK8 GFP pull-down.

**File Name: Supplementary Data 8**

**Description:** Protein groups file, filtered data (processed in R and Perseus) for 12 thousands cells input extract on-chip BUBR1 GFP pull-down on sorted mitotic cells.

**File Name: Supplementary Data 9**

**Description:** Protein groups file, filtered data (processed in R and Perseus) for 4ug and 12 thousands cells input extract on-chip SMC1A GFP pull-down and 8ug and 25 thousands cells input extract on-chip CCDC93 GFP pull-down on final 48- reactors chip.

**File Name: Supplementary Data 10**

**Description:** Protein groups file, filtered data (processed in R and Perseus for 12 thousands cells input extract on-chip Cohesin antibody-based GFP pull-down and 4 µg input extract on-chip Cohesin antibody-based SMC3 pull-down.
