# Supplementary material for: Miniaturised interaction proteomics on a microfluidic platform with ultra-low input requirements
Source: Nat Commun. 2019 Apr 4;10:1525. doi: 10.1038/s41467-019-09533-y (PMC6449397; doi:10.1038/s41467-019-09533-y)
Supplement: Supplementary file 14 — Reporting Summary [file 41467_2019_9533_MOESM14_ESM.pdf]

nature research

Corresponding author(s): Hendrik Marks and Michiel Vermeulen

Reporting Summary

Nature Research wishes to improve the reproducibility of the work that we publish. This form provides structure for consistency and transparency in reporting. For further information on Nature Research policies, see Authors & Referees and the Editorial Policy Checklist.

Statistical parameters

When statistical analyses are reported, confirm that the following items are present in the relevant location (e.g. figure legend, table legend, main text, or Methods section).

n/a	Confirmed	
☐	☒	The exact sample size (*n*) for each experimental group/condition, given as a discrete number and unit of measurement
☐	☒	An indication of whether measurements were taken from distinct samples or whether the same sample was measured repeatedly
☐	☒	The statistical test(s) used AND whether they are one- or two-sided<br>*Only common tests should be described solely by name; describe more complex techniques in the Methods section.*
☒	☐	A description of all covariates tested
☒	☐	A description of any assumptions or corrections, such as tests of normality and adjustment for multiple comparisons
☐	☒	A full description of the statistics including central tendency (e.g. means) or other basic estimates (e.g. regression coefficient) AND variation (e.g. standard deviation) or associated estimates of uncertainty (e.g. confidence intervals)
☐	☒	For null hypothesis testing, the test statistic (e.g. *F*, *t*, *r*) with confidence intervals, effect sizes, degrees of freedom and *P* value noted<br>*Give P values as exact values whenever suitable.*
☒	☐	For Bayesian analysis, information on the choice of priors and Markov chain Monte Carlo settings
☒	☐	For hierarchical and complex designs, identification of the appropriate level for tests and full reporting of outcomes
☒	☐	Estimates of effect sizes (e.g. Cohen's *d*, Pearson's *r*), indicating how they were calculated
☐	☒	Clearly defined error bars<br>*State explicitly what error bars represent (e.g. SD, SE, CI)*

Our web collection on statistics for biologists may be useful.

Software and code

Policy information about availability of computer code

Data collection	Data were collected using a LTQ-Orbitrap Fusion™ Tribrid™ (Thermo Fisher Scientific) running commercial software Xcalibur 4.1 and Tune 3.0.
Data analysis	MS data were analysed by MaxQuant software (version 1.5.1.0) run using standard settings in combination with Andromeda for searches against a SwissProt human database (comprising reviewed entries only and downloaded in June 2017). Perseus software (version 1.5.5.3) and R (version 3.5.1) and Rstudio (version 1.1.456) were used for filtering, analyzing and visualization of the data.

For manuscripts utilizing custom algorithms or software that are central to the research but not yet described in published literature, software must be made available to editors/reviewers upon request. We strongly encourage code deposition in a community repository (e.g. GitHub). See the Nature Research guidelines for submitting code & software for further information.

Data

Policy information about availability of data

All manuscripts must include a data availability statement. This statement should provide the following information, where applicable:

- Accession codes, unique identifiers, or web links for publicly available datasets
- A list of figures that have associated raw data
- A description of any restrictions on data availability

Data are available via ProteomeXchange with identifier PXD012800.

Field-specific reporting

Please select the best fit for your research. If you are not sure, read the appropriate sections before making your selection.

☒ Life sciences ☐ Behavioural & social sciences ☐ Ecological, evolutionary & environmental sciences

For a reference copy of the document with all sections, see nature.com/authors/policies/ReportingSummary-flat.pdf

Life sciences study design

All studies must disclose on these points even when the disclosure is negative.

Sample size	No sample size calculation was done but we used a minimum of 3 replicates per condition as common in label-free MS.
Data exclusions	No data were excluded from the analysis.
Replication	Each experiment was performed in duplicate and each pull-down comprises of three technical replicates for control and test sample.
Randomization	Randomization was performed within the microfluidic platform to ensure there was no positional bias. No other randomization was needed since this is not a population study.
Blinding	No blinding was used since no qualitative data were recorded by the scientists and this is not a clinical study.

Reporting for specific materials, systems and methods

Materials & experimental systems

n/a	Involved in the study	
☒	☐	Unique biological materials
☐	☒	Antibodies
☐	☒	Eukaryotic cell lines
☒	☐	Palaeontology
☒	☐	Animals and other organisms
☒	☐	Human research participants

Methods

n/a	Involved in the study	
☒	☐	ChIP-seq
☒	☐	Flow cytometry
☒	☐	MRI-based neuroimaging

Antibodies

Antibodies used	GFP antibody: Ab6556, Rabbit polyclonal to GFP, Lot.no. GR292567-1, affinity purified form of rabbit polyclonal to GFP (ab290).<br>SMC3 antibody: Ab9263 Anti-SMC3 antibody - ChIP Grade.<br>Rabbit Control IgG (Abcam 46540; Lot.GR63822-2; ChIP grade).<br>Rabbit Control IgG (Santa Cruz sc-2027).<br>GFP Nanobody: LaG-16-2K/R expression vector was obtained from Shi, Y., Pellarin, R., Fridy, P. C., Fernandez-Martinez, J., Thompson, M. K., Li, Y., Wang, Q. J., Sali, A., Rout, M. P., ... Chait, B. T. (2015). A strategy for dissecting the architectures of native macromolecular assemblies. Nature methods, 12(12), 1135-8. LaG-16-2K/R was produced and purified in house as described in the above mentioned paper.
Validation	Ab6556 was validated by western blot by the vendor using extracts from LNCaP transfected with EMPTY vector or pEGFP-PKD1. Primary antibody was used at 1/5000 dilution. Secondary antibody goat anti-rabbit HRP was used at dilution 1/10000. Signal was developed using ECL technique and recorded for 10s. Refer to vendor's web page for results.<br>Ab9263 was validated by western blot by the vendor using Human placenta tissue lysate, K562 and Jurkat whole cell lysate, ICC/

IF: HeLa cells. Human spermatocyte. IP: SMC3 IP in nuclear extracts from HeLa cells. IHC-P: Human colon and testicular seminoma tissue. Refer to vendor's web page for results.
GFP nanobody was originally validated in Fridy PC, et al. A robust pipeline for rapid production of versatile nanobody repertoires. Nature methods. 2014;11:1253–1260.

Eukaryotic cell lines

Policy information about cell lines

Cell line source(s)	HeLa Kyoto and BAC SMC1A and CCDC93-LAP HeLa cell lines were a kind gift of Ina Poser and were produced as described in Poser, I., Sarov, M., Hutchins, J. R., Hériché, J. K., Toyoda, Y., Pozniakovsky, A., Weigl, D., Nitzsche, A., Hegemann, B., Bird, A. W., Pelletier, L., Kittler, R., Hua, S., Naumann, R., Augsburg, M., Sykora, M. M., Hofemeister, H., Zhang, Y., Nasmyth, K., White, K. P., Dietzel, S., Mechtler, K., Durbin, R., Stewart, A. F., Peters, J. M., Buchholz, F., … Hyman, A. A. (2008). BAC TransgeneOmics: a high-throughput method for exploration of protein function in mammals. Nature methods, 5(5), 409-15.
Authentication	None of the cell lines used were authenticated.
Mycoplasma contamination	Cell lines were tested for Mycoplasma contamination upon arrival and were thereafter routinely tested. The cell lines resulted at all points Mycoplasma free.
Commonly misidentified lines (See ICLAC register)	We did not use any commonly misidentified line.
